# Gene Discovery and Advances in Finger Millet [*Eleusine coracana* (L.) Gaertn.] Genomics—An Important Nutri-Cereal of Future

**DOI:** 10.3389/fpls.2016.01634

**Published:** 2016-11-09

**Authors:** Salej Sood, Anil Kumar, B. Kalyana Babu, Vikram S. Gaur, Dinesh Pandey, Lakshmi Kant, Arunava Pattnayak

**Affiliations:** ^1^Indian Council of Agricultural Research, Vivekananda Institute of Hill AgricultureAlmora, India; ^2^Molecular Biology and Genetic Engineering, Govind Ballabh Pant University of Agriculture and TechnologyPantnagar, India; ^3^Indian Council of Agricultural Research, Indian Institute of Oil Palm ResearchPedavegi, India; ^4^College of AgricultureBalaghat, India

**Keywords:** association mapping, blast resistance, genomics, marker assisted selection, millets, transcriptome, transgenics

## Abstract

The rapid strides in molecular marker technologies followed by genomics, and next generation sequencing advancements in three major crops (rice, maize and wheat) of the world have given opportunities for their use in the orphan, but highly valuable future crops, including finger millet [*Eleusine coracana* (L.) Gaertn.]. Finger millet has many special agronomic and nutritional characteristics, which make it an indispensable crop in arid, semi-arid, hilly and tribal areas of India and Africa. The crop has proven its adaptability in harsh conditions and has shown resilience to climate change. The adaptability traits of finger millet have shown the advantage over major cereal grains under stress conditions, revealing it as a storehouse of important genomic resources for crop improvement. Although new technologies for genomic studies are now available, progress in identifying and tapping these important alleles or genes is lacking. RAPDs were the default choice for genetic diversity studies in the crop until the last decade, but the subsequent development of SSRs and comparative genomics paved the way for the marker assisted selection in finger millet. Resistance gene homologs from NBS-LRR region of finger millet for blast and sequence variants for nutritional traits from other cereals have been developed and used invariably. Population structure analysis studies exhibit 2–4 sub-populations in the finger millet gene pool with separate grouping of Indian and exotic genotypes. Recently, the omics technologies have been efficiently applied to understand the nutritional variation, drought tolerance and gene mining. Progress has also occurred with respect to transgenics development. This review presents the current biotechnological advancements along with research gaps and future perspective of genomic research in finger millet.

## Introduction

Finger millet [*Eleusine coracana* (L.) Gaertn.] is a highly self-fertilized allotetraploid (2n = 4x = 36) annual, grown widely as a small grain cereal in arid and semi-arid areas of Central Africa and India. It is a member of the family Poaceae and sub-family Chloridiodeae. It is the only millet belonging to the tribe Chlorideae while all other millets such as *Panicum, Setaria, Pennisetum, Paspalum*, and *Echinochloa* belong to the tribe Paniceae. Finger millet is believed to be one of the few special species that supports the world's food supplies (Vietmeyer et al., 1996). It is a hardy crop that can be grown in diverse environments. The grains can be stored for years without insect damage, which makes it a valuable crop for famine-prone areas. Although grown under dry lands, an assured harvest makes it an indispensable crop in the semi-arid, arid and rainfall limited hill agro-ecosystems. Apart from grains, finger millet provides sufficient straw for animal feeding, which is of great value for backyard animal husbandry in the hills. It is a fairly stress resilient crop and therefore, valuable for contingency crop planning. The grains are a rich source of calcium, iron, dietary fiber, essential amino acids such as methionine, isoleucine, leucine, phenylalanine and are free from gluten. These characteristics indicate that finger millet is not only a food and nutritional security crop of the future, but also is an untapped storehouse of valuable genomic resources, especially for abiotic stresses. However, finger millet is still among many orphan crops lacking genomic information sufficient for use in crop improvement programmes. Efforts to breed finger millet genotypes for developing new cultivars with improved characters have long been tried using traditional plant breeding approaches, but limited success has been achieved. Although traditional plant breeding approaches based on phenotypic selection are very effective, it suffers from several limitations for complex traits such as masking effect of the environment resulting in loss of favorable alleles during selection. The recent advances in omics technologies, i.e., comprehensive and integrated genomics, transcriptomics and proteomics have the potential to elucidate the genetic architecture of plant genomes and decipher the relationship between genotype and phenotype. The rapid advances in DNA sequencing technology have made whole-genome sequencing (WGS) both technically and economically feasible. More than 50 economically important plant genomes have been sequenced and many are underway (Michael and Jackson, 2013).

## Origin and phylogeny

*Eleusine* is a small genus which includes around 9 species distributed in the tropical and subtropical parts of Africa, Asia and South America. The species *E*. *coracana* has two subspecies, *africana* (Kenn.-O'Byrne) Hilu and de Wet and *coracana* (L.) Gaertn. The cultivated species *E. coracana* subsp. *coracana* is an allotetraploid (2n = 4x = 36, AABB) and exhibits morphological similarity to two weedy species, *E. coracana* subsp. *africana* (2n = 36) and *E. indica* (2n = 18). These two weedy species are widely sympatric in Africa, with *E*. *indica* extending to Asia. *E*. *indica* has been confirmed as the maternal diploid genome donor (AA) of *E*. *coracana* subsp. *coracana* as well as *E*. *coracana* subsp. *africana* through cytological studies, isozymes, RAPDs, chloroplast DNA and genomic *in situ* hybridization (GISH) (Chennaveeraiah and Hiremath, 1974; Hilu and de Wet, 1976; Hilu, 1988, 1995; Werth et al., 1994; Bisht and Mukai, 2001). GISH results also suggested *E*. *floccifolia* as the B genome donor to the polyploid species *E*. *coracana* (Bisht and Mukai, 2001). However, Neves et al. (2005) rejected the claim based on nuclear internal transcribed spacers (ITS) and plasmid trnT-trnF sequences. Most likely paternal parent contributing B genome of *E*. *coracana* seems to have become extinct now (Liu et al., 2014).

The cultivated species *E. coracana* subsp. *coracana* includes all cultivated finger millets. Based on inflorescence shape and its correlation with geographic distribution, Hilu and de Wet (1976) recognized three races of cultivated finger millet. Later, five races were identified on the basis of inflorescence morphology in cultivated finger millets (De Wet et al., 1984). Racial evolution in cultivated finger millet species occurred in Africa before this cereal was introduced into India. Race coracana is widely grown across the range of finger millet cultivation in India and Africa and probably gave rise to all other four races of cultivated species through selection under domestication. Races vulgaris, elongata., plana, and compacta may have evolved independently in India and Africa under similar environmental conditions from race coracana. In the cultivated species, little independent racial evolution took place in India (De Wet et al., 1984).

## Germplasm resources

Finger millet has rich diversity, and large collections are conserved in long-term storage in various gene banks across the globe. Indian National gene bank at National Bureau of Plant Genetic Resources (NBPGR), New Delhi holds the largest collection of 10,507 accessions, which includes 117 exotic lines, 7 wild species and rest indigenous lines. A global finger millet collection of 5957 accessions is maintained by the International Crops Research Institute for the Semi-Arid Tropics (ICRISAT), Patancheru, India. This collection contains 105 wild species, 5665 landraces, 137 improved cultivars and 50 breeding lines. Out of which 4585 accessions are of non-Indian origin. USDA in Griffin, Georgia maintains 766 accessions, including 17 wild relatives (*E. floccifolia, E. indica, E. jaegeri, E. multiflora*, and *E. tristachya*). The other countries holding large collections include Kenya (1902), Zimbabwe (1158), Uganda (1155), Nepal (877), Sri Lanka (393), and Bhutan (84) (The Global Crop Diversity Trust, 2012). Germplasm characterization has resulted in the development of a core collection representing ~10% of the world collection (Upadhyaya et al., 2006).

## Molecular markers for assessing genetic diversity, species relationship and origin of finger millet

Genetic diversity refers to the variations within the individual gene loci/among alleles of a gene, or gene combinations, between individual plants or between plant populations. The morphological diversity present within finger millet is immense. For example, a range of seed colors can be produced, which are correlated with protein and calcium content (Vadivoo et al., 1998). Landraces with different attributes (e.g., time to maturity, bird tolerance, drought tolerance) are valued by farmers based on local agricultural complexities that reflect their productivity across multiple agro-economic zones (Tsehaye et al., 2006). Genetic diversity offers opportunities to utilize various genomic resources and technologies to manipulate desirable traits. Studying genetic diversity using molecular markers such as random amplification of polymorphic DNA (RAPDs), inter-simple sequence repeats (ISSRs), simple sequence repeats (SSRs), restriction fragment length polymorphisms (RFLPs), amplified fragment length polymorphisms (AFLPs), and expressed-sequenced tags (ESTs) etc. provide unique opportunities to quickly and accurately access the genetic variability in a given population. Compared to rice and other common cereals, there are very few reports on diversity studies of finger millet using molecular markers. However, the design and use of molecular markers in finger millet genomic studies are now beginning to appear in the literature. Early marker-assisted research suggested that there was little sequence diversity in finger millet populations (Muza et al., 1995; Salimath et al., 1995). Since, finger millet is grown in different parts around the world and is adapted to diverse climatic conditions, the possibility of the existence of considerable genetic diversity among the finger millet germplasm/population cannot be neglected. Subsequent development of molecular markers has enabled linkage map of the finger millet genome to be assembled (Dida et al., 2007). With the recent progress, the availability of a published genomic sequence would accelerate the development of markers to assist genotypic classification and breeding practices.

### Beginning of marker studies

RAPD markers were initially the choice by default to characterize finger millet germplasm. Many efforts made earlier to elucidate the species relationship clearly showed the allotetraploid origin of cultivated species *E*. *coracana* subsp. *coracana*. Direct origin from *E*. *coracana* subsp. *africana* along with *E*. *indica* as one of the genome donors was suggested (Hilu and Johnson, 1992; Hilu, 1995; Salimath et al., 1995). Low level of polymorphism in cultivated finger millet has been reported in most of the studies on diversity analysis using molecular markers (Muza et al., 1995). This could be attributed to narrow genetic pool of cultivated finger millet. However, studies using RAPD markers for genetic diversity observed higher polymorphism ranging from 35 to 100% (Fakrudin et al., 2004; Kalyana Babu et al., 2007; Das and Misra, 2010; Kumari and Pande, 2010; Panwar et al., 2010a; Singh and Kumar, 2010; Ramakrishnan et al., 2015). In all these studies, phenograms based on the RAPD markers data could clearly distinguish the genotypes from different geographical regions as separate clusters. Using multiple markers, Salimath et al. (1995) suggested the use of ISSR markers to distinguish within and between species. However, RAPD markers were reported to detect better polymorphism than ISSR markers in the genetic relatedness study of three varieties of finger millet with seed coat color varying from white to brown (Gupta et al., 2010). The genetic diversity studies using RFLP, RAPD, and ISSR were helpful in species identification, genetic relatedness and clustering of genotypes based on similarity or geographical adaptation. These studies laid the foundation of molecular breeding in finger millet.

### Development and utilization of SSRs

Dida et al. (2007) developed genomic SSRs by isolating di- and trinucleotide SSRs from random genomic *Hind*III, *Pst*I, and *Sal*I libraries of finger millet accession PI 321125 following hybridization of 18,432 double-gridded colonies with mixtures of di [(AG)15/(AC)15] and tri [(AAG)10/(AAC)10/(ATC)10, (AGC)10/(AGG)10, (CCG)10] nucleotides. They identified 82 SSR markers and developed the first skeleton genetic map of finger millet using genomic SSRs (31), RFLP, AFLP, and EST markers. The map spanned 721 cM on the A genome, 787 cM on the B genome and covered all the 18 finger millet chromosomes. Later, the same group (Dida et al., 2008) used 45 genomic SSR markers to evaluate genotypic variation among 79 finger millet accessions belonging to Africa and Asia. This was the first study conducted in terms of an ample number of markers and accessions in this crop. The study predicted the origin of genotypes and also substantiated the theory that finger millet was domesticated in Africa before being introduced to India. In all subsequent studies on genetic diversity, the genomic SSRs developed by Dida et al. (2007) were repeatedly used. Most of these studies were limited to a small set of germplasm collections (Arya et al., 2013; De Villiers et al., 2015), local germplasm of a restricted area (Panwar et al., 2010a,b; Kumar et al., 2012) or the number of markers used were limited where large germplasm sets were analyzed (Bharathi, 2011; Kalyana Babu et al., 2014a, 2016; Ramakrishnan et al., 2016). In one such study using a set of 14 polymorphic genomic SSRs and three genic SSRs (Arya et al., 2013), greater magnitude of diversity in African as compared to Indian finger millet accessions was reported. The same study also reported clustering of South Indian accessions with the African lowland types and North/highland Indian accessions with that of African highland types. These findings could be attributed to geographical adaptation of finger millet genotypes to highlands and lowlands in Africa before its movement to the Indian subcontinent. Moreover, the finger millet cultivars grown in Southern India are morphologically indistinguishable from African lowland types (Hilu and de Wet, 1976). In addition to 82 genomic SSRs developed by Dida et al. (2007), 49 new polymorphic genomic SSRs were developed using the next generation sequencing data by Musia (2013). The genetic variation study among five major species of *Eleusine* [*E. coracana* (*E. coracana* subsp. *coracana* and *E. coracana* subsp. *africana*), *E. intermedia, E. indica, E. multiflora*, and *E. floccifolia*] using genomic SSRs showed highest intra-specific polymorphism for *E. coracana* subsp. *africana* (32.45%), followed by *E. coracana* subsp. *coracana* (16.83%). The results clearly showed that cultivated species, and its immediate progenitor have high genetic polymorphism than the other *Eleusine* species (Dagnachew et al., 2014).

#### EST based SSRs (functional markers)

Microsatellites developed from expressed sequence tags (ESTs), popularly known as EST-SSRs or genic microsatellites, represent functional molecular markers. Putative function for the majority of such markers can be deduced by database searches and other *in silico* approaches. EST-SSR markers are also expected to possess high inter-specific transferability as they belong to relatively conserved genic regions within the genome (Arya et al., 2009). Although, SSRs from EST sequences are very convenient for their ready use in diversity studies, limited availability of EST sequences (1956) so far at NCBI (Gene-bank) database for finger millet has limited the utilization of EST SSRs in finger millet. Characterization of 11 elite germplasm lines of finger millet of Indian and African origin using 31 SSRs from 1740 ESTs (based on di, tri, tetra and penta-nucleotide repeat sequences) gave an amplification product for 17 primer pairs, of which nine were found polymorphic with two alleles per locus (Arya et al., 2009). Similarly, Reddy et al. (2012) mined 132 SSRs and designed 30 SSRs having base length of 20 or more for genetic diversity analysis of 15 finger millet accessions. Only 20 primers showed polymorphism, and 13 primers were identified as having a PIC value above 0.5. Later, Kalyana Babu et al. (2014b) used two search criteria for identification of EST-SSRs. In the first criterion, the minimum number of repeats was 6 for dinucleotide, 4 for trinucleotide, and three for tetra, penta and hexa-nucleotides. In the second criterion, the minimum number of repeats was kept two instead of three for tetra, penta and hexa-nucleotides. Out of 1389 ESTs surveyed, more than two microsatellites were found in 32 EST sequences; other 1357 sequences did not yield any SSR. Dimeric repeat “GA” was the most abundant motif in all the searches, which is almost similar to the reports in other crops (Kantety et al., 2002; Thiel et al., 2003; Senthilvel et al., 2004; Jia et al., 2007). Various studies on the development of genomic and EST SSRs have been listed in Table 1 and the sequence information of polymorphic genomic SSRs is available in the Supplementary File (Table S1).

**Table 1:** **Studies on development of genomic and genic SSRs in finger millet**.

**Study area**	**Markers used**	**Significant results**	**References**
Calcium content-designed from calcium transporters and sensors of rice and sorghum	23 anchored EST SSRs	14 polymorphic markers	Kumar et al., 2015a
Transcriptome analysis of drought tolerance	288 genomic SSRs	32 polymorphic markers	Ramadoss, 2014
Calcium content-designed from calcium transporters and sensors of rice and sorghum	146 EST SSRs	No polymorphism	Yadav et al., 2014
Opaque 2 modifiers- from EST sequences of rice, maize, sorghum; Candidate genes of lysine and tryptophan metabolic pathways	67 EST SSRs (33 EST SSRs and 34 candidate genes based SSRs) and 7 maize genomic SSRs tightly linked to opaque 2 modifier genes	35 polymorphic markers	Kalyana Babu et al., 2014c
Finger and neck blast disease- SSR markers designed from finger millet, rice NBS-LRR region; M. griseae genes of rice; cloned rice blast genes	58 SSRs (43 genic SSRs and 15 rice genomic SSRs tightly linked to blast QTLs)	28 polymorphic markers	Kalyana Babu et al., 2014b,d
Finger and neck blast disease- SSR markers designed from rice NBS-LRR region showing similarity with finger millet EST sequences	13 EST SSRs	8 polymorphic markers	Kalyana Babu et al., 2014e
Opaque 2 modifiers- from EST sequences of rice, maize, sorghum	36 ESTSSRs	15 polymorphic markers	Nirgude et al., 2014
Calcium content- from calmodulin candidate genes viz., Calcium exchangers, channels and ATPases of finger millet, rice, maize, wheat and barley	20 anchored SSRs	5 polymorphic markers	Nirgude et al., 2014
New SSRs from EST sequences of finger millet	45 EST SSRs	3 polymorphic markers	Obidiegwu et al., 2014
New genomic SSRs using *in silico* tools from Roche 454 GS-FLX Titanium sequence data	92 SSRs	49 polymorphic genomic SSRs	Musia, 2013
Development of EST SSRs	3 genic SSRs	-	Arya et al., 2013
Diversity analysis for protein content	24 genomic SSRs	21 polymorphic markers	Kumar et al., 2012
New SSRs from EST sequences of finger millet	30 EST SSRs	20 polymorphic markers	Reddy et al., 2012
Finger and neck blast disease	20 EST SSRs (9 NBS-LRR and 11 EST SSRs)	5 markers were identified to be associated with blast resistance in finger millet	Panwar et al., 2011
Finger and neck blast disease–Conserved region of resistance gene homologs in finger millet using degenerate primers of previous studies	6 RGH specific SSRs	6 markers for blast resistance developed	Reddy et al., 2011
Genetic diversity with respect to calcium content in finger millet	10 genomic SSRs	5 polymorphic SSRs	Panwar et al., 2010b
New SSRs from EST sequences of finger millet	31 EST SSRs	9 polymorphic markers	Arya et al., 2009
Synteny between rice and finger millet	332 loci from 266 primer pairs mapped to 26 LGs highly conserved gene orders between rice and finger millet		Srinivasachary et al., 2007
A genetic linkage map with 131 markers mapped to 16 LGs spanning 721 cM on the A genome and 196 markers mapped to 9 LGs covering 787 cM on the B Genome	82 SSR markers		Dida et al., 2007

### Single nucleotide polymorphism (SNP) identification

Apart from SSRs, two recent studies have also appeared on SNP discovery in finger millet. Kumar et al. (2016b) identified 23,000 SNPs through genotyping by sequencing (GBS) of 113 diverse finger millet genotypes. Similarly, 23285 SNPs were generated using next-generation sequencing of two cultivated finger millet genotypes, and 92 SNP markers were validated further for genetic diversity in cultivated and wild species of finger millet (Gimode et al., 2016). Out of 92, 80 SNP markers were polymorphic. However, SNP markers also resulted in a low PIC value of 0.29 revealing narrow genetic base of finger millet as reported with SSRs (Gimode et al., 2016).

## Comparative genomics applications in the crop

As evident from previous sections that the availability of molecular markers and sequence information is scarce in finger millet, comparative genomics has a greater role in finger millet genome analysis. With the availability of full sequence information of the major cereal crops like rice, maize, and foxtail millet, it is now possible through comparative genomics to identify the genes influencing several important traits in finger millet also. Srinivasachary et al. (2007) made the first attempt to generate a comparative map between rice and finger millet and reported high genomic co-linearity between finger millet and rice. Three finger millet homologous groups, 2, 5, and 6, showed co-linearity with two rice chromosomes each, 2 and 10, 5 and 12, and 6 and 9, respectively. The finger millet—rice comparative maps demonstrated that other than the rearrangements that are necessary to account for the difference in the chromosome number between finger millet and rice, the finger millet genome has remained highly conserved since its divergence from a common ancestor with rice some 60 million years ago. Cross-transferability evaluation of 210 SSR markers from major cereal crops (wheat, rice, maize, and sorghum) to *E. coracana* generated reproducible cross-species or cross-genus amplicons in more than half (57%) of the SSR primers screened (Wang et al., 2005). It was described that the transfer rate of SSR markers was correlated with the phylogenetic relationship or genetic relatedness. Similar studies using foxtail SSRs resulted in consistent amplification with 73–95% cross transferability in finger millet, other millets and non-millet species (Gupta et al., 2012; Pandey et al., 2013; Lata, 2015). This property of cross transferability has been exploited in finger millet for trait based marker identification, particularly for disease resistance and nutritional traits, which is summarized in the subsequent subsections.

### Resistance genes/markers identification for biotic stresses

Blast disease and *Striga* infestation are two important biotic constraints affecting finger millet production. Similar to rice, finger millet blast caused by *Pyricularia grisea* (Cke.) Sacc. is a major disease severely limiting finger millet production and productivity. Efforts have been made to identify and characterize the blast resistance gene analogs using functional molecular markers. Three-fourths of the recognition-dependent disease resistance genes (R-genes) in plants encode nucleotide binding site (NBS) leucine-rich repeat (LRR) domain proteins. Resistance gene homologs using degenerate oligo-nucleotide primers designed from the conserved regions of the nucleotide binding site of the previously cloned plant disease resistance genes have been isolated from finger millet (Panwar et al., 2011; Reddy et al., 2011; Kalyana Babu et al., 2014e; Saha and Rana, 2016) and used for differentiation of blast resistant and susceptible genotypes. Most of the cloned sequences from NBS-LRR region have shown homology to known R-genes, denoted as EcRGHs (*Eleusine coracana* resistance gene homologs) (Saha and Rana, 2016). The EST sequences from NBS-LRR region of finger millet showed homology with rice *PiKh* and *Pi21* blast resistant genes. These results suggested that rice blast resistance gene orthologs may be playing an important role in conferring blast resistance in finger millet (Kalyana Babu et al., 2014e; Kumar et al., 2016a). Few potentially linked SSR markers viz., NBS-5, NBS-9, NBS-7, NBS-3 from the conserved region of NBS LRR and EST-SSRs (EST-SSR4, FMBLEST5) have been identified for blast resistance (Panwar et al., 2011; Kalyana Babu et al., 2014e). The *in silico* comparative analysis of NBS-LRR regions of finger millet with rice showed that the sequences from resistant genotypes had high similarity with the rice blast *Pi* genes and NBS-LRR region. While the sequences obtained from the susceptible genotypes did not find any similarity with the NBS-LRR region (Kalyana Babu et al., 2014e). The sequences obtained from the resistant alleles showed similar areas on the 11th chromosome of rice as also the rice ESTs. This region on the 11th chromosome of rice, therefore, could be playing an important role in the blast disease resistance in finger millet.

*Striga* spp., another major biotic constraint in African countries, which severely affects the crop and yield losses from 20 to 80% have been reported (Atera and Itoh, 2011). Grain yield losses of up to 100% have been reported on susceptible sorghum cultivars under high *Striga* infestation levels (Haussmann et al., 2000). Development and use of resistant cultivars is the most economically feasible and environment-friendly control measure for *Striga*. Stable genetic resistance in adapted genotypes is required for effective control, but *Striga* resistance genes have not been identified in finger millet. The potential sources for *Striga* resistance/tolerance, however, might be available in wild species of finger millet (Kuiper et al., 1998). Some genetic resistance has been reported in rice, sorghum, pearl millet and maize, but no immunity has been identified in any crop (Harahap et al., 1993; Kim et al., 1999; Oswald, 2005; Ejeta and Gressel, 2007). Taking the leads from sorghum, pearl millet, maize, rice and other crops, resistance breeding strategy need to be employed in finger millet, and the identified molecular resources should be deployed to screen finger millet germplasm for *Striga* resistance. Research efforts may also be directed toward the development of herbicide-tolerant genotypes for control of *Striga* infestation in cultivated fields.

### Nutritional traits related genes/markers identification

Finger millet grains are known for its exceptionally high calcium content, 5–30 times higher than the other cereals (National Research Council, 1996). Genetic variation has been reported in finger millet germplasm and grain calcium content as high as 450 mg per 100 g seeds has been reported in some genotypes (Panwar et al., 2010b). In addition, the seeds contain on an average 7% protein, which varies from 4.88 to 15.58% (Singh and Srivastava, 2006), and contains 44.7% of the essential amino acids (Mbithi-Mwikya et al., 2000). A significant negative correlation between calcium and protein content was observed by Kumar et al. (2012) indicating that protein and calcium content improvement programmes cannot be simultaneously taken up in finger millet. Various proteins, including calmodulin, calcineurin-B like proteins, Ca-dependent protein kinases, calreticulin and calsequestrin have been found to regulate the calcium transporters, sensors and responders. Genes encoding these proteins have been speculated to be involved in accumulation of calcium in developing grains of finger millet. The seed storage proteins of cereals are regulated by two important transcription factors, namely, the PBF (Prolamin box binding factor) (Gupta et al., 2011) belonging to the plant specific Dof transcription factor gene family and *Opaque2* (first identified in maize) homologs belonging to the bZIP domain containing transcription factors. *Opaque2* (*o2*) genes play an important role in the regulation of seed storage proteins and *o2* modifiers regulate lysine and tryptophan metabolic pathway enzymes, which results in higher lysine and tryptophan content. The orthologous regions for *o2* modifier genes have been identified in cereal crops such as rice, maize, sorghum, and teff (Onodera et al., 2001). Seeds of some genotypes of finger millet contain high levels of tryptophan compared to other cereals, which indicates genotypic variation in germplasm accessions for this trait. Marker studies have been conducted to identify the alleles for *opaque2* modifiers influencing the tryptophan content in finger millet grains. Nirgude et al. (2014) used 36 EST-SSR primers for the *opaque2* modifiers and 20 anchored-SSR primers for calcium transporters and calmodulin genes for analysis of the genetic diversity of 103 finger millet genotypes for grain protein and calcium contents. Interestingly, the *opaque2* modifier specific EST-SSRs differentiated the finger millet genotypes into high, medium and low protein containing genotypes. However, calcium transporters and calmodulin gene based EST-SSRs broadly differentiated the genotypes based on the calcium content. Later, Kalyana Babu et al. (2014c) developed a set of 67 functional SSRs to investigate the genetic variation in a global collection of 190 finger millet genotypes for *o2* modifiers influencing tryptophan content. The EST sequences included the *opaque2* modifier genes of maize; maize *opaque2* hetero dimerizing protein (OHP), rice *RISBZ* sequences (homologous to maize *opaque*2 sequences), *opaque2* transcription factors, sorghum *opaque*2 modifier genes and 34 genic SSRs from candidate gene of lysine and tryptophan metabolic pathways. These functional SSR loci broadly grouped the global collection of 190 finger millet genotypes into three major clusters (A, B and C) based on tryptophan content. Cluster A comprised genotypes that had medium levels of tryptophan content (0.70–0.85%), while cluster B comprised genotypes containing high tryptophan (>0.85%). Cluster C consisted mostly the genotypes that had tryptophan values of less than 0.70%, with a few exceptions. Few genotypes viz., IE5106, IE6350, GE4449, VHC3903, VHC3870, and GE4811 were identified as having higher tryptophan content of nearly 0.9%.

Utilizing the calcium transporters, calcium binding proteins and calcium-regulated protein kinases genes of rice, sorghum and other Gramineae members, 146 genic SSRs were designed to assay polymorphism and detect marker trait association for calcium content variation in 113 finger millet genotypes with calcium content varying between <100 mg/100 g and 450 mg/100 g grains (Yadav et al., 2014; Kumar et al., 2015a). However, no structural or length polymorphism was observed in the amplicons, indicating a conserved behavior of SSRs among the target candidate genes. The plausible reasons for highly monomorphic behavior across the finger millet genotypes may be that markers derived from protein coding sequences are constrained by purifying selection (Cho et al., 2000; Yadav et al., 2014), and therefore, unable to reveal polymorphism. Another important observation made by Yadav et al. (2014) was that the genic SSRs were successfully cross-transferred at the genus level but could not reveal variations among finger millet genotypes. It was hypothesized that since the mineral transport and the storage machinery largely remained conserved at the intra-specific level, it resulted in the suppression of SSR variations in the genes. High self-pollination in the species, domestication in isolated niche and limited recombination were also suggested as possible reasons for absence of polymorphism in finger millet genotypes. Single nucleotide polymorphism (SNP) in candidate genes, therefore, remains a possible cause of variation in grain calcium contents among finger millet genotypes. Hence, a step forward toward GBS analysis of finger millet genotypes for identification of high density SNP markers linked to grain calcium content was recently taken up by Kumar et al. (2016b), and 23,000 SNPs were identified from 33 GB of sequencing data. In future, these SNPs could be further analyzed to identify useful marker(s) associated with grain calcium and protein content.

### Population structure analysis of the crop

The ability to identify the geographic origin of an individual using biological data poses a formidable challenge in genetics because of its complexity and potentially dangerous misinterpretations (Tishkoff and Kidd, 2004). For instance, owing to the intrinsic nature of biological variation, it is difficult to say where one population ends, and another start by looking at the spatial distribution of traits within the population. Only in the past decade, researchers began harnessing high-throughput genetic data to address this problem. *STRUCTURE* is the widely used software in genetic studies to analyze differences in the distribution of genetic variants among populations with a Bayesian iterative algorithm by placing samples into groups whose members share similar patterns of variation (Pritchard et al., 2000). In case of major cereals, population structure is well studied, but in finger millet, only few studies have been conducted. Dida et al. (2008) studied the population structure of 79 finger millet accessions (*E. coracana*) from 11 African and five Asian countries, including 14 wild *E. coracana* subsp. *africana* lines collected from Uganda and Kenya. They used 45 genomic SSR markers distributed across the finger millet genome. Phylogenetic and population structure analyses showed that the *Eleusine* germplasm formed three largely distinct subpopulations, representing *E*. *coracana* subsp. *africana, E*. *coracana* subsp. *coracana* originating from Africa and *E*. *coracana* subsp. *coracana* originating from Asia. However, Bharathi (2011) found four subpopulations while analyzing large finger millet germplasm of 990 collections. The subpopulation 1 and subpopulation 2 had a mixture of East Africa and South Asian accessions while subpopulation 3 contained maximum number of South Asian accessions, and subpopulation 4 had a mixture of all other geographical origins. Similarly, Kalyana Babu et al. (2014a) studied a subset of the large germplasm representing minicore collection and few Indian genotypes and reported existence of two sub-populations which was in correspondence with the geographical origin of the accessions. However, in the study of Kumar et al. (2015a), the structure analysis did not classify the 113 Asian *coracana* accessions for calcium content variation and distributed them in all four subpopulations without biased distribution toward any particular subpopulation.

**Figure 1:**
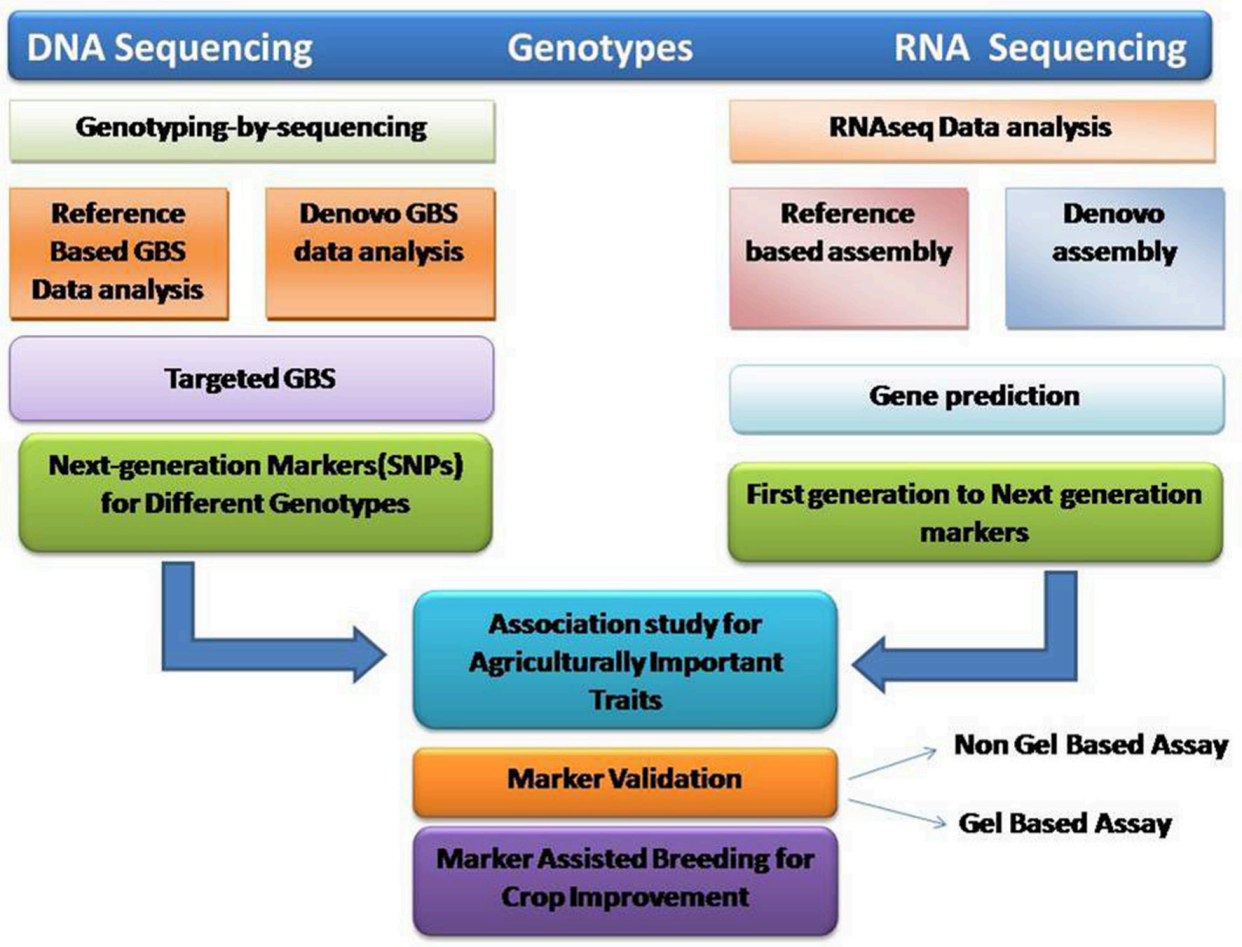
**Use of next generation sequencing technologies based on RNA sequence data and genotype by sequencing for marker discovery**.

Recently, population structure analysis using the SNPs showed three distinctive clusters of accessions, and the population subgroups corresponded largely to geographic regions of accessions with some notable exceptions (Kumar et al., 2016b). Thus, based on structure analysis using molecular markers, clearly finger millet gene pool has 2–4 sub-populations where the Indian and exotic genotypes are separated in different sub-populations with few exceptions, and *Eleusine coracana* subsp. *africana* forms a separate group. Moreover, the separation of accessions into different sub-populations was mainly based on their geographical adaptation (Dida et al., 2008; Bharathi, 2011; Kalyana Babu et al., 2014a). However, in all studies admixtures were observed in the sub-populations. More than 80% exotic alleles in 16 genotypes of the North Western Himalayan region of India and 30% Indian alleles were observed in exotic lines in sub-population 1 (Kalyana Babu et al., 2014a). The occurrence of admixtures could be attributed to *Eleusine* gene flow between India and Africa and higher admixture in Indian or African genotypes probably could be the result of intensive inter-crossing between Indian and African germplasm, a breeding strategy that was adopted long back in India and reciprocated in Africa too. These results are indicative only and more detailed marker studies would be needed to ascertain how the African alleles were distributed across the Indian genotypes. Currently, next-generation sequencing technologies are being used for the development/discovery of next-generation markers and flow chart of marker discovery is given in Figure 1.

## Association mapping of important traits

The rate of genetic enhancement of quantitative traits like grain and fodder yield (and their component traits) is slow because of their complex inheritance, involvement of many physiological processes, and influence of genotype × environment interactions (GEI) (Yadav et al., 2003). Integration of molecular marker technology into a crop breeding programme provides excellent opportunities to deal with these complexities. Most of the work in this area has been directed toward identifying genomic regions of interest to facilitate marker-assisted selection. Although, conventional bi-parental mapping-population-based QTL mapping approach has shown its promise in identifying loci for many important traits in major crop plant species, it is constrained by its low resolution (10–30 cM) (Flint-Garcia et al., 2005) and the limited number of segregating alleles at any locus. Association mapping based on linkage disequilibrium (LD), however, exploits historical recombinations and targets multiple alleles at individual loci to detect marker-phenotype associations to identify genomic regions linked to a wider range of phenotypic traits, and is expected to provide higher resolution (Buckler and Thornsberry, 2002). The availability of plenty of landraces and diverse germplasm of finger millet indicates the possibility to dissect and associate highly useful and agronomically important alleles to traits. However, low level of genetic diversity in *Eleusine coracana* could be the major reason for slow or no progress in this area of finger millet improvement. There is only one report of linkage mapping in finger millet that too with an inter-specific population of *Eleusine coracana* subsp. *coracana* cv. Okhale 1 and its wild progenitor *E*. *coracana* subsp. *africana* accession MD 20 (Dida et al., 2007). Furthermore, there is very little progress toward identification of QTLs for important traits through association mapping in this crop. Association analysis using phenotypic data of agro-morphological traits on finger millet reference set (300 germplasm accessions) and genotypic data of 20 SSR markers revealed 15, 22, and 14 QTLs in three environments and 11 QTLs in a combined analysis. Inconsistent associations between the agronomic traits and markers were mainly due to a limited number of random and non-trait specific markers (Bharathi, 2011). Two markers UGEP8 in LG3 and UGEP56 in LG9 showed a consistent association for days to 50% flowering indicating relatively tight linkage between the trait and marker. Kalyana Babu et al. (2014a) identified four markers UGEP 81, UGEP 77, UGEP 90, and FM 9 tightly linked to QTLs of basal tiller number, days to flowering, flag leaf blade width and plant height, respectively. For blast resistance, 104 SSR markers identified four QTLs for finger blast and one QTL for neck blast resistance (Kalyana Babu et al., 2014b). The rice genomic marker RM 262 and genic marker FMBLEST32 were linked to finger blast disease resistance at a *P*-value of 0.007 and explained a low phenotypic variance of 10 and 8% respectively (Table 2). The genomic marker UGEP81 was associated to finger blast resistance at a *P*-value of 0.009 and explained 7.5% of phenotypic variance. The QTLs for neck blast was associated with the genomic SSR marker UGEP18 at a *P*-value of 0.01 which explained 11% of phenotypic variance (Table 2; Kalyana Babu et al., 2014b). Some loci UGEP56, UGEP8, UGEP65, and UGEP31 had multi trait associations (Bharathi, 2011). UGEP81 was associated with the finger blast resistance as well as with basal tiller number, FM9 had an association with plant height and flag leaf blade width (Table 2). Similarly, multi-markers trait association of UGEP77 and UGEP90 for days to flowering was also observed (Kalyana Babu et al., 2014a). Thus, it is apparent that use of mapping populations to get better idea of consistency of marker association and extensive study of these associated QTL would be useful for confirmation of the multi-trait QTLs identified so far. Both multi-trait single marker and single trait multi-marker associations indicate that quantitative traits are always conferred by multiple loci, and QTLs conferring multiple agronomic traits may cluster around the single regions/markers due to pleiotropic effects of genes.

**Table 2:** **Association mapping studies for identification of linked markers in finger millet**.

**Trait**	**SSR marker/QTLs**	**Probability**	**Phenotypic variance (%)**	**Gene**	**Chromosomal location and distance**	**References**
Calcium content	M2, M6, M11, M16, M26, M27, M36, M45, M65	0.0000, 0.0001, 0.0003	7.9–41.0	Calcium exchangers, calcium channels, calcium ATPase and calcium sensors like calmodulin of cereals (finger millet, rice, maize, wheat and barley)	–	Kumar et al., 2015a
Tryptophan content	OM5, FM8	0.009, 0.004	9.0–11.0	27-kDa c-zein gene of opaque2 modifiers of maize	–	Kalyana Babu et al., 2014c
Protein content	FMO2EST1	0.002	9.0	RISBZ1 gene of rice	–	
Finger blast	RM262	0.007, 0.01	5.0–10.0	Pi-d(t) blast gene of rice	2A (72 cM)	Kalyana Babu et al., 2014b
	FMBLEST32	0.007, 0.01	4.5–8.0	Pi5 blast gene of rice	6B (20 cM)	
	UGEP24	0.003	8.0	–	3B (115.3 cM)	
	UGEP81	0.009	7.5	–	6B	
	UGEP53	0.008	10.5	–	–	
Neck Blast	UGEP18	0.01, 0.009	11.0–13.0	–	1B (70 cM)	
Leaf blast	FMBLEST35	0.009	10.0	Pi21	4B (7.0 cM)	
	RM23842	0.009	11.0	*M*. *grisea*	6B (3.5 cM)	
	FMBLEST15	0.006	8.0	NBS-LRR	4B (6.0 cM)	
Basal tiller number	UGEP81, UGEP1, UGEP8	0.001, 0.017, 0.001	1.4–10.8	–	6B (2.9 cM), 5Ab (25.9 cM), 3B (65.2 cM)	Bharathi, 2011; Kalyana Babu et al., 2014a^^$^^
Days to 50% flowering	UGEP77, UGEP90, UGEP8	0.01, 0.001, 0.001	8.7–58.9	–	3B (4.8 cM), 4B (23.3 cM), 6B (65.2 cM)	
Flag leaf blade width	FM9	0.001	–	–	–	Kalyana Babu et al., 2014a
Plant height	FM9	0.001	11.2–14.1	–	–	
Flag leaf blade length	UGEP31	0.039, 0.004	4.3–95.2	–	3A (75.8 cM), 3B (64 cM)	Bharathi, 2011^^$^^
Finger number	UGEP8	0.013, 0.001	3.06–3.7	–	3B (65.2 cM)	

$ *Only consistent QTLs over environments for the reference set of finger millet were taken*.

Association mapping for nutritional quality trait influencing tryptophan and protein content using 120 (74 genic and 46 genomic) SSR loci resulted in the identification of two linked QTLs for tryptophan and one QTL for protein content (Table 2). The two SSRs associated with the QTLs for tryptophan content were the genic marker OM5 and the genomic marker FM8, explaining 11 and 9% of the phenotypic variance, respectively. The OM5 marker was designed from the 27-kDa c-zein gene of *Opm*, which influences the tryptophan content to a large extent. The QTLs for protein content were associated with the genic SSR marker FMO2EST1, which was designed from the RISBZ1 gene of rice homologous to the *opaque2* modifiers. Interestingly, the protein content showed an inverse relationship with tryptophan content (Kalyana Babu et al., 2014c). The identified locus, therefore, could be a down-regulator of *o2* modifier genes. The 220 bp allele of SSR locus OM5 was found to be associated with high tryptophan containing genotypes particularly exotic genotypes and North Western Himalayan genotypes of Indian Sub-continent (Kalyana Babu et al., 2014c). Being an important nutritional trait in finger millet, efforts were also made to identify linked markers for calcium variation in the finger millet grains, and nine markers were found to be associated with calcium content in finger millet grains (Kumar et al., 2015a; Table 2). However, the number of studies and the material used do not exhibit a progress in marker assisted selection for important traits in the crop. There is a greater need of involving diverse germplasm, good phenotyping facilities and genotyping with appropriate number and choice of molecular markers to identify robust markers, especially for blast resistance and nutritional quality traits. Once the markers linked to the QTLs for traits of interest are identified, they can be further used for fine mapping, map based cloning of the full-length genes and in marker assisted breeding for introgression of alleles into locally well adapted genotypes.

Gene homologs of agronomically important traits in major cereals may assist with targeted breeding efforts in crops that are less characterized. Specifically, sequence variants of these genes may be used to develop orthologous molecular markers. Those variants that correlate with desired traits may be used to screen accessions and subsequently assist in marker-assisted breeding efforts. For example, finger millet researchers have isolated orthologs of genes known to be involved in grain amino acid composition (*Opaque2*) and calcium content (calcium transporters, calmodulin) (Reddy et al., 2011; Nirgude et al., 2014). The researchers then associated SSR polymorphisms within these genes to characterize accessions that differed in their protein and calcium content, thus creating a targeted, cost-effective crop improvement strategy. A similar strategy to improve finger millet seed calcium content was also reported independently that focused on orthologs of calcium-binding proteins (CBPs) with extensive characterization of a seed dominant calmodulin (Kumar et al., 2014a, 2015b). A parallel strategy has been suggested for disease resistance in finger millet based on the initial isolation of disease resistance receptors (Reddy et al., 2011; Kalyana Babu et al., 2014b).

## Omics revolution through hi-throughput technologies

**Figure 2:**
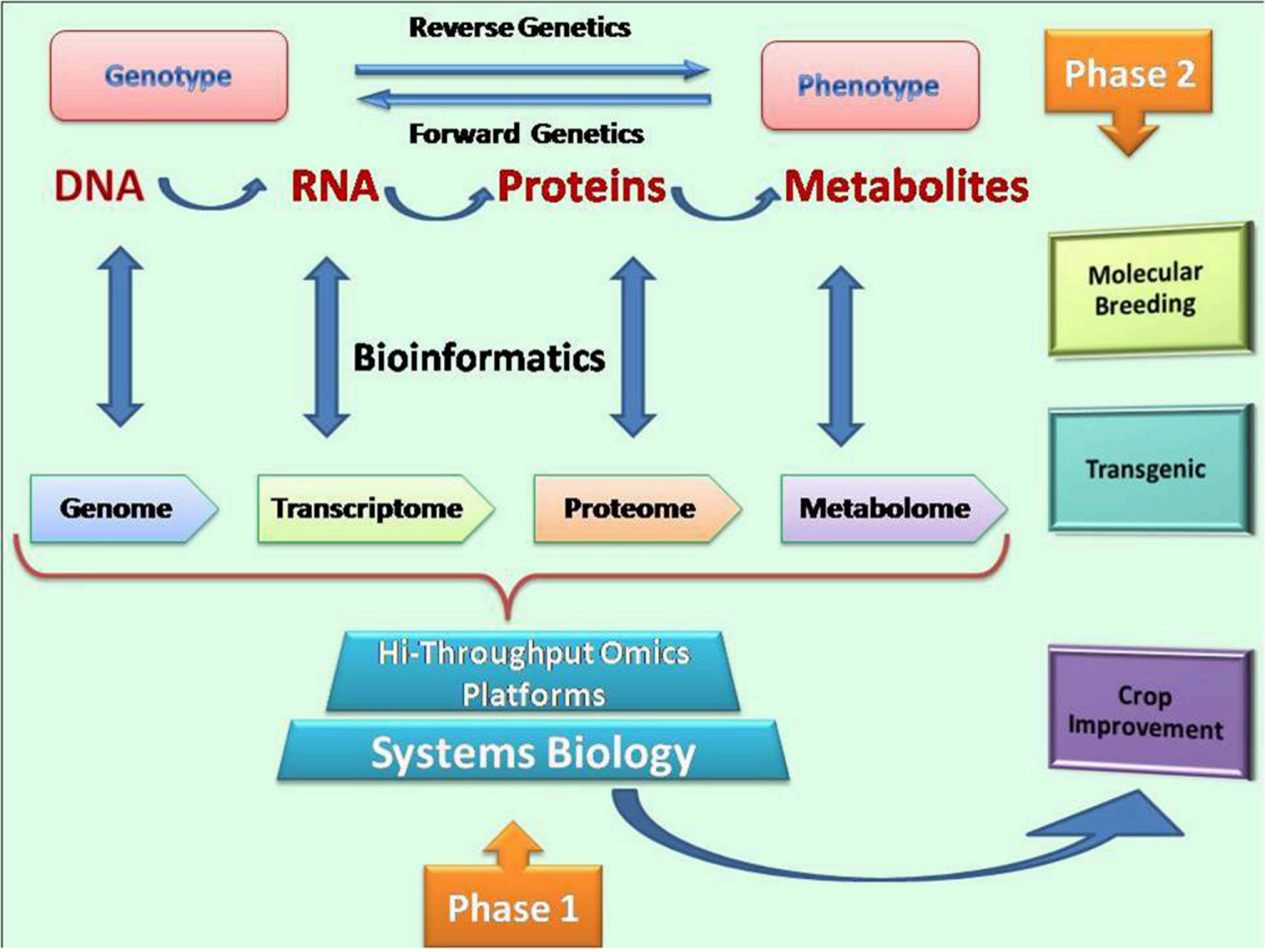
**Omics approaches for crop improvement: filling the gaps between genotype and phenotype in order to resolve agriculturally important complex traits**.

Finger millet is bestowed with agriculturally and nutritionally important traits such as its adaptive nature, good ability to grow under organic conditions and high mineral content (calcium, iron and zinc) and quality proteins. A revolution in the field of omics science will help in understanding the complexity of these traits. The aim of functional genomics is to discover the function of all genes, typically through high-throughput approaches such as genomics, proteomics or metabolomics combined with bioinformatics tools for data analysis and functional analysis. Discovery of gene functions is an essential task in functional genomics; however, it is not sufficient for crop improvement and probably of little use for enhancing selection for quantitative traits such as crop yield. To decode agronomical traits, functional genomics approaches can be of good use for understanding molecular and genetic processes underlying complex traits. Functional genomics approaches such as transcriptomics, proteomics and metabolomics should be combined with environmental and physiological information for gene identification that aims to improve complex traits in plants. An often emerging problem is the lack of correlation between protein and mRNA abundance levels in plants. The changes induced in protein expression do not correspond linearly to the changes in mRNA expression. Furthermore, it has been shown mathematically that a detailed understanding of the control of even the simplest gene network requires information at both mRNA and protein expression levels. Thus, there is a clear need for an integrated approach through blending of functional genomics, proteomics and metabolomics parameters for understanding the biological systems. Thus, a new branch of biology that aims to discover and understand biological properties emerging from the interactions of many system elements is termed as “Systems biology.” Systems biology approaches facilitate a multi targeted approach by allowing identification of regulatory hubs in complex networks. It works on molecular parts (transcripts, proteins and metabolites) of an organism to fit them into functional networks. The major reason why systems biology is gaining interest today is that progress in molecular biology, particularly in genomics and proteomics is enabling scientists to collect comprehensive data on a wide variety of plant traits. The power of these new tools would lead to an explosion of information unparalleled in the history of biology and can also be exploited for unraveling the complex traits of agriculturally and nutritionally important finger millet crop by following hi-throughput omics platforms (Figure 2).

### Gene expression and transcriptome analysis

Finger millet is not only known for its superior tolerance against various abiotic stresses such as drought and salinity but also for its high nutritional value. Research endeavors to address these important traits using modern technologies such as high-throughput transcriptome sequencing is now beginning to appear (Table 3). Rahman et al. (2014) identified several salinity responsive genes and genotype specific responses from transcriptome analysis of contrasting finger millet genotypes differing for salinity tolerance through mapping and annotation of identified transcripts using rice gene models. Using qRT-PCR, Nageshbabu et al. (2013) showed the expression change of 12 conserved miRNAs in 8 days old finger millet seedlings exposed to salt and drought stress. Of the 12 miRNAs, eight responded to both salt and drought stress where the miRNA response toward salt stress was tissue specific, a majority of abiotic stress specific miRNAs were transcription factors such as MYB-Protein, AHAP2, NF-Yα 2, CCAAT binding protein and other zinc finger proteins involved in transcription machinery (Nageshbabu et al., 2013).

**Table 3:** **Transcriptome analysis in finger millet**.

**Tissue**	**Sequencing platform**	**Genotypes/traits**	**References**
Four stages of Pooled developing spikes	Illumina HiSeq 2000 platform	Transcriptome sequencing of two contrasting finger millet genotypes for calcium content	Kumar et al., 2015b
Pooled developing spikes	Illumina HiSeq 2000 platform	Transcriptome sequencing of two finger millet genotypes differing in grain calcium content	Singh et al., 2014
Leaves	Ion Proton platform	Sequencing of salinity responsive leaf transcriptome of two contrasting finger millet genotypes	Rahman et al., 2014

As the crop is valued for its high calcium content, studies have been focused to characterize calcium sensing, transport and accumulation mechanisms across genotypes differing in their grain calcium content with the use of high-throughput transcriptome sequencing. The finger millet plant might as well befall as a model system for better understanding of the underlying genetic control and molecular physiological mechanisms contributing to high grain calcium. To increase the nucleotide sequence information resource and for gaining deep insights about the developing grain, Kumar et al. (2015b) used the short reads of Illumina HiSeq-2000 high-throughput RNA sequencing technology to sequence the entire developing seed transcriptome of two finger millet genotypes differing in grain calcium content (GPHCPB 45, a high calcium genotype, and GPHCPB 1, a low calcium genotype). Mirza et al. (2014) hypothesized that finger millet variably accumulates calcium in different tissues due to differential expression of genes involved in uptake, translocation and accumulation of calcium. Hence, expression profiles of Ca^2+^/H^+^antiporter (CAX1), two pore channel (TPC1), CaM-stimulated type IIB Ca^2+^ATPase and two CaM dependent protein kinase (CaMK1 and 2) homologs were studied in finger millet. Further studies on finger millet CAX1 and ATPase might offer new insights in understanding their role in high calcium accumulation during seed development. Through systems biology approach, 82 unique calcium sensor genes identified from finger millet transcriptome data have been categorized into eight calcium sensor gene families viz., CaM and CaMLs, CBLs, CIPKs, PEPRKs, CRKs, CDPKs, CaMKs, and CCaMK. Out of 82 genes, 12 were found to be different from the rice orthologs (Singh et al., 2014; Kumar et al., 2015b). Nineteen Ca^2+^ transporter genes identified from developing seed transcriptome data of finger millet showed 33–90% variation in amino acid sequence compared to rice Ca^2+^ transporter (Kumar et al., 2015c). High level of correlation between the expression of “EcCAX3” a calcium exchanger gene and the amount of calcium accumulated in spikes could be utilized in a future biofortification program (Singh et al., 2014; Singh U. M. et al., 2015). Further attempts have also been made to identify and characterize the calcium binding proteins (CaBPs) in grain filling stages of finger millet using proteomics, bioinformatics and molecular approaches.

Similar to the high grain calcium content trait of finger millet, research endeavors were also focussed to investigate the mechanisms behind its impressive high nitrogen use efficiency (NUE) trait. Gupta et al. (2011), studied the effects of nitrogen inputs on growth, yield and NUE components along with the activities of important enzymes involved in nitrogen uptake (nitrate reductase-NR) and ammonium assimilation (glutamine synthetase-GS, glutamine oxoglutarate aminotrasferase -GOGAT and glutamate dehydrogenase-GDH) at different developmental stages. Correlation studies indicated that grain protein content an important grain quality trait was negatively correlated with NUE and nitrogen utilization efficiency but positively correlated with nitrogen uptake efficiency. NUE, on the other hand, was positively correlated with nitrogen utilization efficiency, but negatively correlated with nitrogen uptake efficiency. To further understand the molecular basis of high NUE of finger millet, five genes (EcHNRT2, EcLNRT1, EcNADH-NR, EcGS, and EcFd-GOGAT) involved in nitrogen uptake and assimilation were isolated using RACE (Rapid Amplification of cDNA Ends) and conserved primer approaches. Time kinetics experiment using nitrogen starved finger millet seedlings revealed that all the five genes except EcHNRT2 in the leaves of GE3885 (High-protein genotype) were induced within 30 min of nitrate exposure indicating quick transportation of nitrate signals to the leaves in response to a nitrogen deficit in leaves.

Furthermore, compared to the low-protein genotype, expression of HNRT2 was strongly induced in both roots and shoots of high-protein genotype at the least nitrate concentration supplied. These experiments indicated that the high seed protein finger millet genotypes are conceivably quick sensors of soil nitrogen compared to the low-protein genotypes. Expression of EcDof1 was also found to overlap the expression of NR, GS, and GOGAT indicating that Dof1 probably regulates the expression of these genes under different conditions by sensing the nitrogen fluctuations around the root zone (Gupta et al., 2013). Another study indicated that the expression of EcDof1 in higher grain protein genotype was more consistent with the expression of carbon metabolism genes, suggesting that EcDof1 differentially regulates the expression of these genes and simultaneously controls the grain protein content in finger millet genotypes (Kanwal et al., 2014). Recently, expression profiling of 10 full-length Dof genes in two genotypes of finger millet (GE1437 and GE3885) differing in seed protein content revealed their differential expression in all stages of plant development. Some of the Dof genes were found to express preferentially during grain filling and seed development process indicating their important roles during seed development such as regulating the accumulation of carbohydrates and protein. The behavior of Dof1 and Dof2 which simultaneously regulate the genes involved in nitrogen and carbon metabolism has also been analyzed. The Dof1 transcription factor is an activator while the Dof2 acts like a repressor. Both the proteins interact with the same target DNA sequence in the promoter. Gupta et al. (2014) reported that in the roots of a high-seed protein genotype the *EcDof1*/*EcDof2* ratio was greater than that of a low-protein genotype indicating a higher activation of N uptake and assimilation genes (Gupta et al., 2014). The authors suggested that this ratio might be utilized to screen finger millet genotypes for high NUE.

### Proteomics approaches

The term proteome can be applied to a complete set of proteins in a given organism or a specific subset of proteins present in a particular cell type or under specific growth conditions. Proteomic analysis is another approach that has been used to characterize the molecular components of heterosis and thus, will be helpful in crop improvement. The field of proteomics has helped researchers to understand the effects of proteins on plant mineral nutrient homeostasis. It seeks to monitor the protein fluctuations under variable developmental and environmental influences, as programmed by the genome, and mediated by the transcriptome. Singh U. M. et al. (2015) made efforts to characterize calcium binding proteins in order to validate their function not only from the viewpoint of plant calcium nutrition, but also their functional role in calcium uptake and bioavailability using proteomics platforms involving 2D electrophoresis and MALDI-TOF peptide mass fingerprinting (PMF) techniques. Calcineurin-B and calreticulin like proteins have been identified through different techniques, including Stains-all, calcium overlay assay and PMF in the seeds of finger millet. These proteins are probably involved in retaining high calcium content in the finger millet seeds (Singh U. M. et al., 2015). To understand the mechanism involved in calcium accumulation during finger millet seed development a calmodulin (CaM) gene that is strongly expressed during developing spikes of high grain calcium genotype was identified. Using immunodetection techniques it was revealed that CaM is localized in the embryo and aleurone layer and accumulates in higher amounts in high grain calcium genotype compared to the low grain calcium genotype (Kumar et al., 2014a). It would be tempting to identify the calcium ATPases and other proteins expressed specifically in developing seeds that interact with CaM. These proteins once fully characterized can be used in preparing food supplements rich in calcium.

The seed storage protein genes and their regulatory components which make finger millet superior to other cereals and millets have not been investigated so far. The main reason behind it is lack of conserved sequences among seed storage proteins across cereals and genomic information on finger millet. Major seed storage proteins of finger millet are prolamins which account for approximately half of the total grain nitrogen. They are further characterized as alpha-prolamin, beta-prolamin, gamma-prolamin and delta-prolamin types, out of which alpha-type prolamins appear to be the major components and constitute about 70% of total prolamins (Kumar et al., 2014b). The seeds of finger millet are not only a good source of high quality proteins containing essential amino acids like lysine and methionine, but also a rich source of vitamins like thiamine, riboflavin, and niacin.

## Transgenic development

The basic prerequisites for transgenic development are the availability of potential candidate genes, and an efficient regeneration and transformation protocol, which can be deployed not only in finger millet, i.e., development of cisgenics but also in other staple cereal crops for augmentation of yield and nutritional quality. The advent of genomics has played a pivotal role in the identification of superior genes for crop improvement, and such approaches are also being tried to identify agriculturally and nutritionally important genes from finger millet. Progress has also occurred with respect to transgenic protocols for finger millet utilizing *Agrobacterium* and callus cell bombardment approaches (Kothari et al., 2005; Ceasar and Ignacimuthu, 2009, 2011; Sharma et al., 2011; Jagga-Chugh et al., 2012; Plaza-Wüthrich and Tadele, 2012). Latha et al. (2005) first developed transgenic finger millet plants expressing *pin* gene and demonstrated that the expression of fungicidal PIN protein of prawn in the transgenic finger millet bestows high-level of resistance against *Pyricularia grisea*. Similarly, a serine-rich-protein (PcSrp) encoding gene isolated from the cDNA library of salt stressed roots of *Porteresia coarctata* expressed in finger millet improved salt tolerance of finger millet (Mahalaksmi et al., 2006). They showed that transgenic plants expressing PcSrp gene were able to grow to maturity and set seed under 250 mM NaCl stress, whereas control plants failed to survive under similar salt stress. Ceasar and Ignacimuthu (2011) proved the amenability of finger millet to Agrobacterium-mediated gene transfer and developed leaf blast resistant transgenic finger millet plants using rice chtinase gene (Ignacimuthu and Ceasar, 2012; Table 4). Transgenic finger millet plants expressing the mannitol biosynthetic pathway gene mannitol-1-phosphate dehydrogenase (*mtlD*) from bacteria were developed through *Agrobacterium tumefaciens*-mediated genetic transformation (Hema et al., 2014). The transgenic plants showed better growth under drought and salinity stress compared to wild-type. In order to understand the mechanisms that enable the plants to cope with drought stress, Singh R. K. et al. (2015) established the role of drought-tolerant finger millet EcDehydrin7 protein in transgenic tobacco. They also observed relatively high expression of EcDehydrin7 protein compared to wild type in biochemical assays as well as qRT PCR analyses of transgenic tobacco plants, which clearly envisaged that finger millet EcDehydrin7 protein can be used in other food crops for development of drought-tolerant transgenic. Transgenic approaches have allowed finger millet plants to be improved for disease, drought and salt/salinity tolerance (Ramegowda et al., 2012; Anjaneyulu et al., 2014; Hema et al., 2014), zinc accumulation (Cakmak, 2008), and disease resistance (Latha et al., 2005).

**Table 4:** **Transgenic development efforts in finger millet for stress tolerance**.

**Trait**	**Transformation method**	**Gene/protein**	**References**
Leaf blast	Particle gun	Pin gene (pPin 35S) bar gene (pBar 35S)	Latha et al., 2005
Salt tolerance	Particle gun	*Porteresia coarctata* serine-rich-protein (PcSrp) encoding gene	Mahalaksmi et al., 2006
Leaf blast disease	Agrobacterium mediated	Rice chitinase gene	Ceasar and Ignacimuthu, 2011; Ignacimuthu and Ceasar, 2012
Various abiotic stresses including drought and salt	Agrobacterium mediated	A stress responsive NAC gene (*EcNAC1*) from finger millet was expressed in tobacco	Ramegowda et al., 2012
Salt tolerance	Agrobacterium mediated	Vacuolar proton pyrophosphatase gene (SbVPPase) from Sorghum bicolor	Anjaneyulu et al., 2014
Drought tolerance	Agrobacterium mediated	EcDehydrin7 protein of finger millet expressed in tobacco	Singh R. K. et al., 2015

## Genome size and sequencing initiatives

Analyses of nuclear DNA content in the species of *Eleusine* were first carried out by Hiremath and Salimath (1991) using Feulgen microspectrophotometry. The 2C DNA amount in the diploid species ranged from 2.50 pg in *E. verticillata* to 3.35 pg in *E. intermedia*. In contrast, the tetraploid species showed a range from 4.95 pg in *E. coracana* subsp. *africana* to 6.13 pg in *E. floccifolia*. Polyploid species showed approximately double the genome size of that of their corresponding diploids indicating an evolutionary increase in DNA amount in *E. coracana* during the course of its origin and domestication. Later Mysore and Baird (1997) using laser flow cytometry, determined the genomic size of *E. coracana* as 2C = 3.36–3.87 pg (2n = 36), (1 pg = 980 Mbp). Recently, the finger millet genome size has been measured as 1.8 pg/1C nucleus by flow cytometry (Dida et al., 2007). Gupta and Ranjekar (1981) found that ~39–49% of the genome of finger millet consists of repetitive DNA sequences, which intersperse with 18% of single copy DNA sequences of 1900 nucleotide pairs. In order to unravel this complex, large genome of finger millet, two independent programmes on whole genome sequencing started in the year 2014. The Bio-Resources Innovations Network for Eastern Africa Development (Bio-Innovate) program initiated the finger millet genome sequencing project to complement the work on identifying, developing and delivering millet varieties to smallholder farmers in the Eastern Africa region (Goron and Raizada, 2015). Bio-Innovate has partnered with the African Orphan Crop Consortium to sequence the finger millet genome. This initiative is being coordinated by the International Crops Research Institute for the Semi-Arid-Tropics (ICRISAT) regional team based in Nairobi in partnership with Biosciences Eastern and Central Africa (BecA) Hub, University of California, University of Georgia (UGA) and the Swedish University of Agricultural Sciences (SLU) (http://www.bioinnovate-africa.org/finger-millet-genomics-project-to-provide-researchers-with-better-tools-for-variety-production/). The second one is Indo Swiss collaborative programme funded by Department of Biotechnology, Ministry of Science and Technology, India on “Genetic Enhancement and Bioavailability of Finger Millet” that aims to enhance the yield potential and bioavailability of essential nutrients in finger millet. The project is being coordinated by University of Agricultural Sciences, Bangalore, India in partnership with Functional Genomics Center Zurich (FGCZ), University of Zurich. In addition to whole genome sequencing and transcriptome analysis, the project also aims to identify genotypes with reduced anti-nutritional factors to enhance the bio-availability of essential nutrients (http://www.fgcz.ch/research/Finger_Millet.html). Genome sequencing will provide finger millet breeders a map that can be used to easily identify and locate genes responsible for progressive traits in finger millet genotypes to assist the breeding process. *Eragrostis tef*, a member of the finger millet subfamily Chloridoideae again an orphan tetraploid (2n = 4x = 40) has been recently sequenced and could serve as reference till the finger millet genome is sequenced (Cannarozzi et al., 2014).

## Research gaps and challenges

Although conventional breeding efforts have made significant progress in the development of new improved high yielding varieties of the crop in India and Africa, systematic efforts to understand the genetics, physiology and molecular basis of abiotic stress tolerance and nutritional traits variation are still in infancy. There is large gap in germplasm characterization and utilization in breeding programmes as wide phenotypic diversity is not reflected at genetic level due to low genetic polymorphism. Low genetic polymorphism has posed as a major challenge in molecular mapping progress, as a result only single linkage map with few markers is available in the crop. In all major crops, wild relatives have been well characterized and utilized to transfer many important traits including disease resistance, however, finger millet wild relatives have not been utilized so far. Further, the number and type of molecular markers availability in the crop are another concern that requires concerted efforts to develop more number of microsatellites and SNPs to target important traits in the crop. Reliable and precise information on QTLs/genes governing important target traits, i.e., blast and striga resistance, drought tolerance and high NUE is lacking in the crop. Even the alleles/genes for nutritional traits due to which the crop has regained its lost importance are also not known. These traits require development of large diverse phenotyping panels in addition to more number of molecular markers saturating the crop genome for valid marker trait associations.

## Way forward

Genomic information has not only helped in the understanding of structural and functional aspects of many plant genomes but also provided a feasible platform for manipulation of genomes for crop improvement. In recent years, sequence information has become readily available for a variety of crop species, but minor crops such as finger millet still lag behind. With the advent of next generation sequencing technologies it is now relatively easy to generate sufficient data for computational gene and molecular marker discovery. Linking or correlating markers with heritable traits can be used to associate the genotype with the expressed phenotype. This has the potential to develop millions of novel markers and potential candidate genes in finger millet. Once large number of molecular markers are available, genomic selection will be the appropriate strategy for stacking beneficial QTL alleles for complex traits with low heritability. Until then, comparative genomics is an opportunity to use sequence variants of important genes of major cereals and other millets to develop orthologous molecular markers, and the markers that correlate with target traits can be used to screen large panel of accessions.

## Author contributions

SS conceptualized, wrote and critically revised the manuscript for publication. AK added information and edited the manuscript. BK and VG assisted in writing. DP, LK, and AP edited the manuscript.

### Conflict of interest statement

The authors declare that the research was conducted in the absence of any commercial or financial relationships that could be construed as a potential conflict of interest.
